# Acute Cognitive Performance and Mood Effects of Coffeeberry Extract: A Randomized, Double Blind, Placebo-Controlled Crossover Study in Healthy Humans

**DOI:** 10.3390/nu15112418

**Published:** 2023-05-23

**Authors:** Philippa A. Jackson, Charlotte Kenney, Joanne Forster, Ellen F. Smith, Rian Elcoate, Bethany Spittlehouse, Jodee Johnson, David O. Kennedy

**Affiliations:** 1 Brain Performance and Nutrition Research Centre, Northumbria University, Newcastle upon Tyne NE1 8ST, UK; charlotte2.kenney@northumbria.ac.uk (C.K.); jo.forster@northumbria.ac.uk (J.F.); ellen.f.smith@northumbria.ac.uk (E.F.S.); david.kennedy@northumbria.ac.uk (D.O.K.); 2 Health Research Authority, Holland Drive, Newcastle upon Tyne NE2 4NQ, UK; bethany.spittlehouse@hra.nhs.uk; 3 PepsiCo, Health & Nutrition Sciences, Chicago, IL 60607, USA; jodee.johnson@pepsico.com

**Keywords:** coffeeberry, polyphenols, cognition, mood

## Abstract

Background: Coffeeberry extract, rich in chlorogenic acids, shows promise in improving mood and cognition, particularly when co-supplemented with phenolic compounds. However, limited work has considered the effects of coffeeberry in isolation, especially at low doses. Objective: The current study investigated the effect of low and moderate doses of coffeeberry extract on cognition and mood. Design: This randomized, double-blind, placebo-controlled crossover design investigated three active beverages on a sample of 72 healthy adults aged 18–49 years. The investigational beverages contained 100 mg or 300 mg coffeeberry extract (standardized to 40% chlorogenic acid), or 75 mg caffeine (positive control). Cognition, mood, and subjective energy were measured at baseline and then again at 60 and 120 min post-treatment. Results: Analysis revealed no effect of 300 mg coffeeberry extract, while 100 mg resulted in increased mental fatigue during the performance of cognitively demanding tasks (*p* = 0.025) and decreased accuracy on a task of sustained attention (*p* = 0.003), compared to placebo, at 60 min post dose. Conclusions: Administration of 100 mg and 300 mg coffeeberry extracts revealed limited, transient negative effects following 100 mg coffeeberry. Given the large number of outcome measures analysed and the absence of findings following the 300 mg dose, these negative findings should be interpreted with caution. Overall, the findings of the current study suggest that coffeeberry extract at a low or moderate dose does not have a beneficial effect on mood, mental and physical energy levels, or cognition; higher doses, as have been administered previously, may be more effective.

## 1. Introduction

Considerable interest has been placed on the potential health-promoting effects of polyphenols, specifically with regards to their effects on cognition. [1,2]. Here, acute supplementation with phenolic-rich extracts shows cautious promise in improving aspects of cognitive performance [3,4,5,6] and mood [7,8].

Coffeeberry extract (also referred to as coffee cherry), rich in chlorogenic acids (CGA) and low in naturally occurring caffeine, is a promising candidate. Previous work has indicated that coffeeberry extract can interact with biological systems underlying brain function. For example, acute supplementation of 100 mg whole coffee fruit extract has been shown to significantly increase concentrations of brain-derived neurotropic factor (BDNF) in plasma [9]. Given the importance of this protein in memory and learning processes, the ability for coffeeberry to modulate circulating BDNF shows promise for improving cognitive performance. Further, coffeeberry extract is rich in polyphenols, specifically CGA, which have also been shown to have beneficial effects on cognition and mood [10,11,12], although duration and treatment composition may be important factors with regards to efficacy. With regards to potential mechanisms, Kwon et al. [13] revealed that CGA inhibited acetylcholinesterase activity in the hippocampus and frontal cortex and inhibited scopolamine-induced memory impairment in a dose-dependent manner up to 9 mg/kg CGA in mice. Similarly, Bouayed et al. [14] demonstrated the anxiolytic effects of 20 mg/kg CGA in rodent models of anxiety that were subsequently blocked by the administration of flumazenil, suggesting effects of CGA at benzodiazepine receptors.

To date, investigations of coffeeberry extract have explored effects following single doses of coffeeberry and also when co-supplemented with additional phenolic and herbal compounds. Taking the latter as an example, in healthy, young adults, acute improvements in mood were observed following 1100 mg coffeeberry extract (440 mg chlorogenic acid, 22 mg caffeine) in combination with beetroot (10 g), ginseng (170 mg), and sage (280 mg) extracts [15]. Here, reductions in confusion, fatigue, and total mood disturbance were also observed, as measured by the Profile of Mood States (POMS), as well as lower mental fatigue and increased alertness during the performance of cognitively demanding tasks, which the authors ascribed to the chlorogenic acid content of the extract. Despite clear mood enhancements and increased oxygenated haemoglobin in the prefrontal cortex, no improvements were observed on cognitive task performance. In contrast, when presented in combination with different herbal extracts (300 mg *Bacopa monnieri* and 100 mg *Panax quinquefolius ginseng*), acute supplementation of 100 mg whole coffee fruit extract (40 mg chlorogenic acid) improved working memory task performance in healthy adults [16]. Additionally, concomitant changes in hemodynamic response (reductions in activation in the pre-frontal cortex) were observed. In both examples, the combination of herbal extracts rendered the individual effects of coffeeberry on mood and cognitive performance difficult to establish, particularly given the evidence for these herbal supplements to interact with brain function and related mechanisms [17,18,19].

Trials administering coffeeberry extract in isolation to cognitively intact adults are limited; however, similar mood enhancements have been observed following 1100 mg coffeeberry extract [20]. Here, participants reported reduced fatigue/inertia and increased vigour/activity as assessed by POMS, and increased alertness before and during the performance of cognitively demanding tasks. In terms of cognitive performance measures, improved accuracy on the Rapid Visual Information Processing (RVIP) task was observed following coffeeberry extract. The authors suggest that collectively, these findings indicate a pattern of increased engagement following coffeeberry. Similarly, following low (100 mg) and moderate (300 mg) doses, coffeeberry resulted in reduced subjective fatigue and increased alertness during completion of a cognitively demanding battery [21]. Here, an effect of delayed word recall accuracy was observed whereby an attenuated decline in performance was seen following 100 mg coffeeberry compared to placebo at 120 min post dose. Taken together, these findings suggest coffeeberry extract at varying doses may exert beneficial effects on mood and cognitive performance in healthy adults. In order to strengthen the knowledge base regarding the effects of coffeeberry extract and confirm that the previous results are reproducible, the current study aimed to replicate these aforementioned findings by investigating the effects of low (100 mg) and moderate (300 mg) doses of chlorogenic acid rich coffeeberry extract on cognition and mood.

## 2. Materials and Methods

### 2.1. Study Design and Participants

The study followed a randomised, double-blind, placebo-controlled crossover design with four study arms. Research took place from July 2021 to January 2022 at the Brain, Performance and Nutrition Research Centre (BPNRC) at Northumbria University. The Northumbria University Health and Life Sciences Ethics Committee reviewed and approved all study procedures used (Reference 33646), with investigations following the rules of the Declaration of Helsinki 1975. The study was also pre-registered on clinicaltrials.gov (Reference NCT04975802).

**Figure 1:**
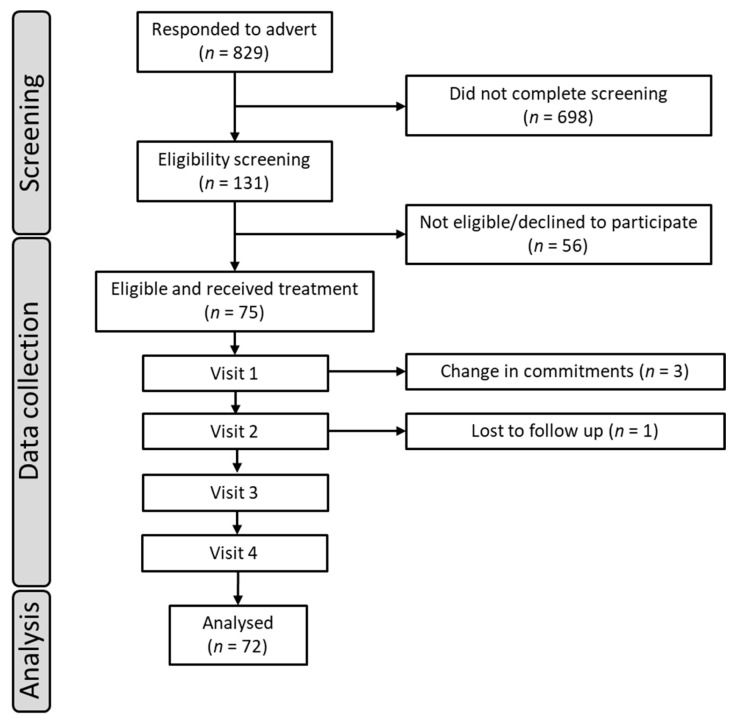
Participant disposition flowchart.

A volunteer sample of 75 participants aged 18–49 years was recruited and consumed at least one dose of treatment. Three of these participants discontinued with the study following the first testing visit, with a further participant discontinuing following the second testing visit. Three of these participants were replaced, with one not replaced due to time constraints. The data from the unreplaced participant were still included within the final analysis, leaving a total sample of 72 participants. For a full summary of enrolment and flow of participants, see Figure 1. Participants self-reported to be in good health, free of chronic illness, have a BMI within the range of 18.5–30 kg/m^2^, have normal or corrected-to-normal vision, and speak English fluently or as a first language. Further details on inclusion and exclusion criteria can be found within the Supplementary Materials. Physiological measurements (height, weight, waist-to-hip ratio (WHR), and blood pressure) were taken upon participants attending the laboratory; all other exclusion criteria were self-reported by participants. Participant characteristics at enrolment can be found in the Supplementary Materials.

A power calculation based upon previous repeated measures data showing an effect size of f = 0.175 [21] indicated that 68 participants would allow detection of significant effects with a power of 0.8 at α = 0.05 in a repeated measures design involving four treatment arms and two assessments. The correlation across time was assumed to be r = 0.5. The estimate was rounded up to 72 to allow for a balanced crossover design.

### 2.2. Treatments

When attending each of the four testing visits, participants were given one of four beverages (labelled A, B, C, D) via random allocation according to a counterbalancing order (Latin square) that was generated by computer. Specifically, participants were block randomized to one of eight possible treatment orders (ABCD, ADCB, BADC, BDCA, CADB, CBAD, DABC, DCBA); participants who replaced a discontinued participant were allocated to the same treatment order as the participant they replaced. The bottles contained 10 oz and were identified only by their letter indicator.

The beverages were matched for colour, aroma, and taste, with a cherry flavour being selected to mask any differences in taste that may have been apparent. The placebo beverage, sweetened with sucralose (0.03%), contained no active ingredients. The treatment beverages contained the placebo drink as a base, along with either 100 mg coffeeberry extract, 300 mg coffeeberry extract, or 75 mg caffeine acting as a positive control due to being an established psychostimulant. The coffeeberry extract was standardized to 40% chlorogenic acid (CognatiQ^®^, FutureCeuticals, Momence, IL, USA).

### 2.3. Cognitive and Mood Assessment

The cognitive function tests were administered to participants using the Computerised Mental Performance Assessment System (COMPASS, Northumbria University, Newcastle upon Tyne, UK). COMPASS allows for bespoke task batteries to be designed as needed, with randomised parallel versions of the tasks being administered at each of the assessments for each participant.

The cognitive assessment administered to participants was primarily comprised of four repetitions of the Cognitive Demand Battery (CDB), a ten-minute battery of three tasks plus two visual analogue scale (VAS) ratings of alertness and mental fatigue. The CDB consists of serial subtractions of both 3 s and 7 s, along with a sustained attention task (rapid visual information processing (RVIP)); full descriptors of these tasks can be found within the Supplementary Materials. Nutritional interventions have been found to influence the results of tasks that require a high cognitive load over an extended period, such as CDB, in previous research [3,20,22,23,24].

As previous research involving extracts containing chlorogenic acid has found them to modulate episodic memory [15,25,26], the following tasks were also incorporated into the assessment: delayed word recall, delayed word recognition, and delayed picture recognition. Administering these tasks though COMPASS has indicated that they are sensitive to nutritional interventions [15,17,27,28,29]. Full descriptors of these tasks can be found within the Supplementary Materials.

Mood and psychological state was assessed with Bond–Lader visual analogue scales [30] and mental and physical energy and fatigue scales (EFS-State scales) [31]; both are well-validated measures of mood. The Bond–Lader mood scales are comprised of 16 lines anchored by antonyms at either side (e.g., ‘tranquil’ and ‘troubled’). Participants are asked to indicate how they are currently feeling on the line between the two words, with ratings scored as a percentage from left to right. The 16 scales are condensed into three scores, as derived from a factor analysis, namely, ‘alertness’, ‘contentedness’, and ‘calmness’. The EFS-State scales ask participants to rate their current subjective feelings of physical energy, physical fatigue, mental energy, and mental fatigue; each of the dimensions is composed of three items that are amalgamated to create a score from 0 to 300 for that dimension.

### 2.4. Procedure

Participants were required to attend six appointments: a remote screening appointment, a training appointment, and four test visits. The remote screening visit was conducted over the telephone, and it comprised a briefing on the requirements of the study, the signing of a virtual consent form, review of the inclusion and exclusion criteria included in the self-reported health screening, completion of the Caffeine Consumption Questionnaire (CCQ), and collection of relevant demographic information such as handedness. Following the initial remote screening, eligible participants were then required to attend a training visit, in which a physical informed consent form was signed. Physiological information that could not be collected remotely, namely, height, weight, waist-to-hip ratio (WHR), and blood pressure (BP), was collected, along with a measure of their usual sleep. Training on the cognitive tasks, EFS, and mood scales was also completed; the completion of cognitive training ensured that participants understood the instructions for both the tasks and the testing procedures and allowed individuals who were unable or unwilling to perform to a minimum standard to be excluded. The first testing visit was at least 48 h and no more than 27 days after the training visit, with each testing visit being 7 days apart (mean 8.55 days ± 5.01; range 5–45 days). Reasons for date deviations more than 14 days were related to COVID-19 infection and/or testing visits falling over the Christmas holiday period.

**Figure 2:**
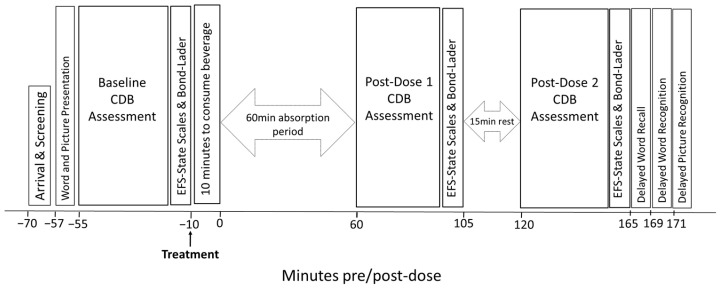
Timeline of the testing visit assessment schedule. CDB, Cognitive Demand Battery; EFS-State, mental and physical energy and fatigue scales.

On each of the four testing visits, participants attended the laboratory having followed the pre-visit instructions given at the training visit specifically (a) to consume the same meal no later than an hour before each visit; (b) to avoid caffeine in any form for 12 h prior to the visit; (c) to avoid moderate-to-vigorous physical activity for 24 h prior to the visit; (d) to have a usual night sleep prior to each visit (no more than ±1.5 h according to the self-reported amount at the training visit); and (e) to avoid alcohol for 24 h prior to the visit). Participants first completed the baseline testing battery (picture and word memory task presentation, 4 repetitions of CDB, followed by EFS and mood scales) before being given their assigned treatment beverage and consuming it fully within 10 min. A 60-minute absorption rest period followed, during which participants were instructed to refrain from mobile phone use but were allowed to watch TV or read. At 60 min post-treatment, participants completed the testing battery of four repetitions of CDB and EFS and mood scales again before being given another quiet rest period of 15 min. At 120 min post-treatment, participants completed the testing battery a final time (four repetitions of CDB and EFS and mood scales), along with completing the episodic memory tasks. All testing visits were identical aside from the randomly generated parallel versions of each of the memory tasks and lasted 4–4.5 h in length (Figure 2). Participants received GBP 200 upon completion of the study, or pro rata for withdrawals as requested.

### 2.5. Statistics

All cognitive and mood outcome measures were modelled using the MIXED procedure in SPSS (version 26.0, IBM Corp., Armonk, NY, USA). For the episodic memory outcomes, the post dose data were modelled including treatment as a fixed effect. For the CDB task and VAS outcomes, changes from baseline values were modelled with treatment, minutes post dose (60, 120), repetition (1–4), and their interactions as fixed effects and participant as a random effect. For the psychological state and mood scales, changes from baseline data were modelled with treatment, minutes post dose (60, 120), and their interaction as fixed effects and participant as a random effect. Pairwise comparisons were all specified a priori and carried out using Sidak corrections. The a priori comparisons of 100 mg and 300 mg coffeeberry against placebo at 120 min post dose on the mental fatigue and alertness scales were specified as primary endpoints.

## 3. Results

Of the seventy-five participants who were randomized to receive treatment, three participants discontinued following testing visit 1, and one discontinued following testing visit 2. Of these four participants who discontinued, three were replaced and one could not be replaced due to time constraints of the study timeline. However, their data were included in the final analysis, leaving a final dataset comprising 72 participants. Approximately half (*n* = 35) reported regular consumption of either normal or decaffeinated coffee beverages; caffeine consumption was 114.21 mg/d ± 104.81 (range 0–500 mg/d) across the whole sample. Seventeen adverse events were reported during the supplementation period; however, all were ‘unrelated to the treatment’ (headache, aches and pains, common cold, COVID-19, sore throat, tiredness).

### 3.1. Response to Coffeeberry

#### 3.1.1. Mood and Psychological State

For the primary endpoints, a priori pairwise comparisons of change from baseline data collected at 120 min post dose revealed no significant effect of treatment on either the mental fatigue or alertness scales (CDB).

**Figure 3:**
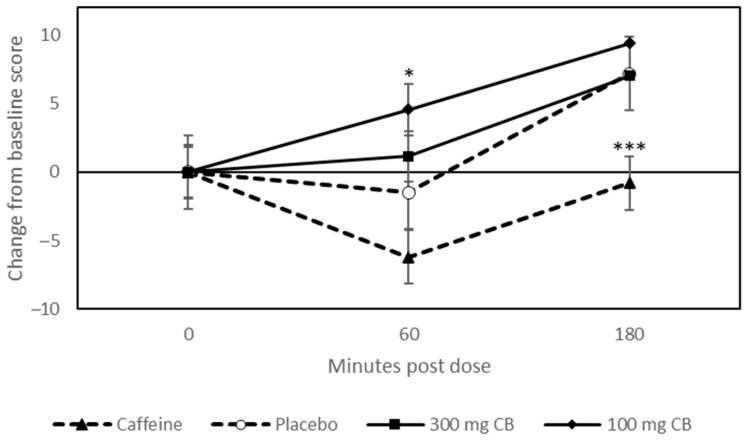
Estimated marginal means of change from baseline values (±SE) derived from the linear mixed model for the Cognitive Demand Battery (CDB) ‘Mental Fatigue’ Visual Analogue Scale (VAS). Pairwise comparisons revealed that when participants consumed 100 mg CB, they felt more mentally fatigued (*p* = 0.025). Significance tests are pairwise comparisons of the estimated marginal means corrected for multiple testing (Sidak). Letters denote significant difference between placebo and specified beverage. * *p* < 0.05; *** *p* < 0.001.

A priori pairwise comparisons of change from baseline data collected at 60 min post dose revealed the mental fatigue (CDB) was significantly increased following 100 mg coffeeberry, compared to placebo (*p* = 0.025) Figure 3. All other a priori comparisons for mood and psychological state were non-significant.

No other mood or psychological state effects were observed, including mental fatigue on the EFS.

#### 3.1.2. Cognitive Performance

**Figure 4:**
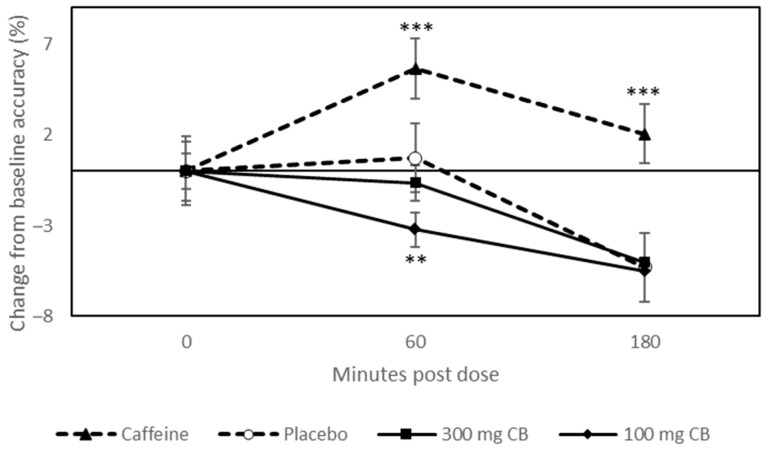
Estimated marginal means of change from baseline values (±SE) derived from the linear mixed model for accuracy performance on the Rapid Visual Information Processing (RVIP) task. Pairwise comparisons revealed that when participants consumed 100 mg CB, RVIP accuracy was significantly reduced at 60 min post dose (*p* = 0.003). Significance tests are pairwise comparisons of the estimated marginal means corrected for multiple testing (Sidak). Letters denote significant difference between placebo and specified beverage. ** *p* < 0.01; *** *p* < 0.001.

A priori pairwise comparisons of change from baseline data, collected at 60 and 120 min post dose, revealed that 100 mg coffeeberry significantly decreased RVIP accuracy at 60 min post dose compared to placebo (*p* = 0.003) (see Figure 4).

No other significant comparisons were observed. See Supplementary Materials for the results of the analysis for all study outcomes.

### 3.2. Response to Caffeine (Positive Control)

The analysis revealed that of the 25 outcome measures that were collected at each of the two post dose assessments, a significant main effect of treatment was observed for 24 of these. Pairwise comparisons revealed a consistent alerting and performance enhancing effect of the positive control beverage containing 75 mg caffeine in comparison to both the placebo beverage and the drinks containing coffeeberry, although there was some variation in where the significant comparisons were observed (see Supplementary Materials). No effect of treatment was observed for any of the delayed episodic memory tasks. Overall, the pattern of effects confirmed that the study procedures and participants were sensitive to a known psychostimulant.

## 4. Discussion

The results of the current study suggest that the consumption of 300 mg coffeeberry extract has no effect on mood, performance of cognitively demanding tasks, or episodic memory up to 120 min post dose. Limited results were observed following 100 mg coffeeberry, which followed a pattern of negative effects. Specifically, compared to placebo, participants who received 100 mg coffeeberry reported increased mental fatigue during performance of cognitively demanding tasks at 60 min post dose, although no effect was observed for mental fatigue on the EFS-State. In addition, decreased accuracy on the RVIP task was also observed following 100 mg coffeeberry at 60 min post dose compared to placebo. In contrast to this, a consistent pattern of alerting and performance enhancing effects was observed following 75 mg caffeine compared to placebo—and in some instances also compared to the two coffeeberry beverages—confirming that the study procedures were sensitive, despite the null or negative effects observed following the coffeeberry interventions.

The findings of the present study differ from those reported in previous intervention trials investigating coffeeberry extract either co-supplemented with other phenolics or administered in isolation. Specifically, the negative results following 100 mg coffeeberry extract observed here are unexpected and difficult to explain; however, when considering the current literature, there is some disparity in findings at this dosage. Previously, 100 mg of the same coffeeberry extract that was evaluated in the current study co-administered with 275 mg apple extract had no effect on performance of the CDB (also with four repetitions per assessment) or measures of episodic memory and executive function assessed at 60-, 180- and 360-min post dose [20]. Conversely, when co-supplemented with 300 mg *Bacopa monnieri* and 100 mg *Panax quinquefolius* ginseng, 100 mg whole coffee extract improved working memory performance (on 2-back and 3-back tasks) at 45 min post dose [16]. Similarly, a small pilot study in older adults with subjective cognitive decline reported that single doses of 100 mg coffeeberry extract improved reaction times on both an attention and working memory task at 90 min post dose, which was coupled with neurofunctional changes indicative of improved efficiency [32]. Although the authors’ conclusions were based on within-group comparisons only, these results suggest that lower doses may be effective in populations suffering from cognitive decline.

The current study did not replicate the findings of Reed et al. [21], who implemented similar study procedures to those described here. These authors reported an increase in alertness and decrease in mental fatigue at 120 min post dose following 100 mg coffeeberry and similar effects following the 300 mg dose as well. Whilst both studies implemented similar study procedures, it is difficult to directly compare their respective outcomes as the statistical approaches applied were different, with our group employing a linear mixed model approach that included baseline performance as a covariate and subject as a random effect to improve estimates. An additional key difference between these trials is that the present study employed a considerably larger sample size (*n* = 72 vs. *n* = 30). However, whilst this does not offer an explanation for the differing effects observed following coffeeberry, the increased statistical power here allowed us to confirm the expected alerting and performance-enhancing effects of 75 mg caffeine, which Reed et al. failed to demonstrate. One possible explanation may be associated with the coffee-consuming status of the study samples. In the present study, just under half of the participants reported consuming coffee beverages. Although these data were not presented in the Reed study, it is likely that coffee consumption was more prevalent in their sample, given that consumption of coffee is higher in the USA compared to the UK [33]. Microbial differences have been observed in faecal samples from habitual consumers and non-consumers of coffee, demonstrating the long-term effects of coffee consumption on the gut microbiome [34]. This finding suggests that regular consumers of coffee may be better able to metabolize the chlorogenic acids and other polyphenols contained in the coffeeberry extract, given that the mutual relationship between polyphenol intake and the gut microbiome increases polyphenol bioavailability [35]. Enhanced bioavailability in regular consumers of coffee may therefore limit conclusions when comparing findings across studies with potentially different levels of background coffee consumption. Whether this is relevant across the short post dose period measured in both these studies is unknown. Regardless, future interventions with coffeeberry extract should therefore always measure and report the regular coffee consumption of participants.

One consideration with regards to the negative findings is that it could be hypothesized in a dose–response model that any effects, positive or negative, would be exaggerated following three times the dose. However, this was not observed in the current study, with no effects observed following 300 mg coffeeberry. Therefore, given the small number and transient nature of these negative effects amongst many analyses, and the fact that this is the first report of any negative effects on cognitive performance or mood measures following interventions containing coffeeberry extract [15,16,20,21,36], these results should be viewed with caution and require further investigation.

Previously, data from our research centre has shown a consistent alerting/arousing effect of 1100 mg coffeeberry extract when administered in isolation [20] and in combination with other botanical extracts [15] up to 6 h post dose. Taken together with the findings of the present study suggests that the lowest effective dose of coffeeberry exists somewhere between 301 and 1099 mg and should be identified in future studies. Previous studies have revealed that ~500 mg total polyphenols is an effective dose across a range of parameters including cardiovascular function [37] as well as brain function and behaviour for other polyphenols, including resveratrol [38], anthocyanins (blackcurrant) [39], and cocoa flavanols [3]. The coffeeberry extract used in the present study contained at least 40% chlorogenic acids (CGA); given that 1100 mg coffeeberry extract contains around 440 mg CGA, it is possible that this may already be close to the lowest effective dose in healthy populations. Indeed, previous evidence has observed improved sustained attention and alertness following acute supplementation with a decaffeinated green coffee blend rich in chlorogenic acids (532 mg) and low in caffeine (5 mg) [10].

Besides dose, a final consideration is the timing of the assessments, which only extended until 120 min post dose in the current study. Bioavailability data suggest that a number of CGA only reach peak concentration in plasma after 4 and 6 h post dose [40], which presents the possibility that the assessment period in the current study may have been too short if some of the previously observed effects are underpinned by CGA metabolized in the colon. Therefore, future dose ranging studies should include assessments up to at least 6 h pose dose.

## 5. Conclusions

In summary, an assessment of the effects of administration with either 100 or 300 mg coffeeberry extract at 60- and 120-min post dose on mood and performance during and following a battery of cognitively demanding tasks revealed limited, transient negative effects following 100 mg coffeeberry. Given the large number of outcome measures analysed and an absence of effects following the 300 mg dose, caution should be applied in interpreting these results. Overall, the findings of the current study suggest that coffeeberry extract at a low or moderate dose does not have any effect on mood, mental and physical energy levels, episodic memory, or cognitively demanding tasks up to 120 min post dose.

## Data Availability

The data presented in this study are available upon request from the corresponding author. The data are not publicly available due to data sharing and privacy regulations in Europe and UK.

## Supplementary Materials

The following supporting information can be downloaded at: https://www.mdpi.com/article/10.3390/nu15112418/s1, Table S1: Participant characteristics at enrolment for continuous variables. N = 72; Table S2: Participant characteristics at enrolment for categorical variables. N = 72. Table S3: Estimated means ± standard error of the mean (SEM) derived from linear mixed models of change from baseline data for outcome measures where the Type III tests of fixed effects showed a significant treatment effect. *p* Values are derived from pairwise comparisons between caffeine and the three other investigational beverages using Sidak adjustment for multiple comparisons. Table S4: Performance on the cognitive demand battery task outcomes. The Baseline data are raw means (plus SEM) collected prior to dosing. Data presented from the 60 and 120 minute post dose (p.d.) assessments are estimated means (plus SEM) of change from baseline values derived from the linear mixed model analysis, with the final two columns showing test statistics (F) and associated probabilities (p) for each of the fixed effects included in the model. Table S5: Scores for the Energy and Fatigue and Bond-Lader mood scales. The Baseline data are raw means (plus SEM) collected prior to dosing. Data presented from the 60 and 120 minute post dose (p.d.) assessments are estimated means (plus SEM) of change from baseline values derived from the linear mixed model analysis, with the final two columns showing test statistics (F) and associated probabilities (p) for each of the fixed effects included in the model. Table S6: Scores for the episodic memory tasks, completed following the 120 min post dose assessment. Data are estimated means (plus SEM) derived from the linear mixed model analysis, with the final two columns showing test statistics (F) and associated probabilities (p) for the effect of treatment. References [3,23,41,42,43,44] are cited in the supplementary materials.
