# Supplementary material for: Acute Cognitive Performance and Mood Effects of Coffeeberry Extract: A Randomized, Double Blind, Placebo-Controlled Crossover Study in Healthy Humans
Source: Nutrients. 2023 May 23;15(11):2418. doi: 10.3390/nu15112418 (PMC10254646; doi:10.3390/nu15112418)
Supplement: Supplementary file 1 [file nutrients-15-02418-s001.zip › nutrients-2384642-supplementary.pdf]

**Supplementary Materials**

1. **Eligibility criteria**

**Inclusion criteria**

- Participants are aged 18–49 years, inclusive [1]
- Participants self-report that they are in good health
- Willing to maintain habitual diet, physical activity pattern, and body weight throughout the trial
- Willing to abstain from consumption of caffeine within 12 h of testing
- Willing to abstain from alcohol consumption and avoid vigorous physical activity for 24 h prior to all test visits
- Willing to refrain from 'over the counter' medications (e.g. pain medication) and stimulant medication for 12 h, seasonal allergy/hayfever nasal antihistamine medications for 24 hours and oral antihistamines for 48 h prior to all test visits
- Understanding the study procedures and willing to provide informed consent to participate in the study and authorization to release relevant protected health information to the study Investigator.

[1] Age range was based on research demonstrating natural decline in some cognitive performance measures is more likely to occur at 50+ years in the general population

**Exclusion criteria**

Participants complying with at least one of the following criteria were not eligible:

- Failure to meet any one of the inclusion criteria
- Current use of prescription medication, except for contraceptives
- Report hypersensitivity to caffeine
- Major trauma or major surgical event within 6 months of screening
- Extreme dietary habits, as judged by the Investigator (high fat, very high protein diets, intermittent fasting, etc.)
- Exposure to coffeeberry within 30 d prior to screening
- History of cancer in the prior two years, except for non-melanoma skin cancer
- Have a visual impairment that cannot be corrected with glasses or contact lenses
- Food allergies/intolerances/sensitivities to any ingredients in the study products (including coffee or related foods/beverages/products)
- Self-report excessive leisure time physical activity (>7 strenuous bouts per week)
- Have a current or chronic gastrointestinal, sleep, or psychiatric disorders
- Work night shifts or follow a variable work pattern that results in irregular sleep pattern
- Are pregnant, trying to get pregnant or lactating
- Smoke tobacco, vape nicotine or use nicotine replacement products
- Use illegal/recreational drugs
- Unable to demonstrate adequate minimal performance on lab, computer-based cognitive tasks (See Appendix V for minimum performance scores)
- Have participated in another clinical trial within past 30 days and/or participation in another PepsiCo trial in the past 6 months
- Have high blood pressure (systolic over 139 mm Hg or diastolic over 89 mm Hg)
- Have a Body Mass Index (BMI) outside of the range 18.5–35 kg/m$^2$

- Have learning and/or behavioural disorders such as dyslexia or ADHD
- Excessive caffeine intake (>500 mg per day)
- Have taken dietary supplements e.g. Vitamins, omega 3 fish oils etc. in the last 4 weeks (Note: participation is possible following a 4 week supplement washout prior to participating and for the duration of the study on the proviso that the supplements they are taking are out of choice and not medically prescribed or advised)
- Have any health condition that would prevent fulfilment of the study requirements (this includes non-diagnosed conditions for which no medication may be taken)
- Has been diagnosed with/ undergoing treatment for alcohol or drug abuse in the last 12 months
- Have been diagnosed with/ undergoing treatment for a psychiatric disorder in the last 12 months
- Suffers from frequent migraines that require medication (more than or equal to 1 per month)
- Any known active infections
- Does not have a bank account (required for payment)

## 2. Participant characteristics at enrolment

**Table S1.** Participant characteristics at enrolment for continuous variables. N = 72.

| Measure | Mean | SD |
|---|---|---|
| Age (years) | 28.85 | 8.45 |
| Education (years) | 17.47 | 2.81 |
| Normal hours sleep per night | 7.64 | 0.68 |
| Fruit & veg consumption (portions/day) | 3.39 | 1.72 |
| Caffeine consumption (mg/day) | 114.21 | 104.81 |
| Systolic BP (mm/Hg) | 119.03 | 11.678 |
| Diastolic BP (mm/Hg) | 77.58 | 7.34 |
| Heart Rate (beats per minute) | 75.14 | 13.45 |
| BMI (kg/m$^2$) | 24.873 | 3.52 |

**Table S2.** Participant characteristics at enrolment for categorical variables. N = 72.

| Measure | Count | % |
|---|---|---|
| Male/female | 26/46 | 36/64 |
| Race: | | |
| White | 50 | 69.4 |
| Black | 3 | 15.3 |
| Asian | 11 | 11.1 |
| Other | 8 | 4.2 |
| Wear glasses (yes/no) | 31/41 | 43/57 |
| Handedness (left/right) | 7/65 | 10/90 |
| Dietary habits: | | |
| Omnivore | 62 | 86 |

| | | |
|---|---|---|
| Vegetarian | 8 | 11 |
| Pescatarian | 2 | 3 |
| Highest level of education: | | |
| Entry Level (Entry Level Certificates) | 0 | 0 |
| Level 1 (NVQ level 1, GCSE D-G, BTEC Intro) | 0 | 0 |
| Level 2 (NVQ level 2, GCSE A*-C, Young Apprenticeships, BTEC ½) | 4 | 5.6 |
| Level 3 (NVQ level 3, AS & A-levels, BTEC 3) | 19 | 26.4 |
| Level 5 (NVQ level 5, HND, HNC, Dip HE/FE, Found. Deg, BTEC 5) | 4 | 5.6 |
| Level 6 (Bachelors Degree, GradDip, Grad Cert., BTEC 6) | 29 | 40.3 |
| Level 7 (Masters, PGDip, PG Cert, BTEC 7) | 14 | 19.4 |
| Level 8 (Doctorates, BTEC 8) | 2 | 2.8 |

## 3. Computerised cognitive tasks

### Cognitive Demand Battery (CDB)

The objective of this battery is to assess the impact of treatment on speed/accuracy and mental fatigue during continuous performance of cognitively demanding tasks.

Participants complete the 10-min battery of tasks four times in immediate succession (i.e., for a continuous period of 40 min).

Application of this battery has been shown to reliably increase self-ratings of 'mental fatigue' and to be sensitive to a number of herbal and natural interventions [1–5]. Particularly relevant here, the battery has been shown to be sensitive to performance enhancement by single doses of cocoa-flavanols (520 mg/920 mg) [6] and caffeinated products [1,2].

The 10-min battery comprises:

*Serial 3s subtraction task* (2 min): Computerised versions of the serial subtraction tasks will be implemented using tests of 2-min duration. Participants are required to count backwards in threes from a given number as quickly and as accurately as possible using the number keys to enter each response. A random starting number between 800 and 999 is presented on the computer screen, which is cleared by the entry of the first response. The task is scored for number of correct responses and number of errors. In the case of incorrect responses subsequent responses are scored as positive if they were scored as correct in relation to the new number.

*Serial 7s subtraction task* (2 min): This is identical to the serial threes task with the exception that it involves the serial subtraction of sevens.

*Rapid Visual Information Processing task* (RVIP – 5 mins): The participant is required to monitor a continuous series of digits for targets of three consecutive odd or three consecutive even digits. The digits are presented at the rate of 100 per minute and the participant responds to the detection of a target string by pressing the response button as quickly as possible. The task is

continuous and lasts for 5 minutes, with 8 correct target strings being presented in each minute. The task is scored for percentage of target strings correctly detected, average reaction time for correct detections, and number of false alarms.

'*Mental fatigue' visual analogue scale*; Participants rate their current subjective 'mental fatigue' state by making a mark on a line with the end points labelled "not at all" (left hand end) and "extremely" (right hand end). Scores are calculated as percentage along the line from left to right.

*'Alertness' visual analogue scale*; Participants rate their current subjective 'alertness' state by making a mark on a line with the end points labelled "not at all" (left hand end) and "extremely" (right hand end). Scores are calculated as percentage along the line from left to right

*'Motivation' visual analogue scale*; Participants rate their current subjective level of 'motivation' by making a mark on a line with the end points labelled "no motivation at all" (left hand end) and "strongest degree of motivation imaginable" (right hand end). Scores are calculated as percentage along the line from left to right

**Episodic memory**

*Word Presentation*: A unique set of fifteen words will be presented. Words will be selected at random from a large bank of words (MRC Psycholinguistic Database) matched for word length, frequency, familiarity and concreteness. Stimulus duration will be one second, as will be the inter-stimulus duration.

*Picture presentation*: Fifteen colour photographic images of objects will be presented sequentially on screen for the participant to remember at the rate of 1 every 1 s, with a stimulus duration of three seconds.

*Delayed Word Recall:* Participants write down as many of the 15 words that they were presented during the stimulus presentation period. This task is scored for accuracy only.

*Delayed Word Recognition*: A series of words are displayed on the screen, one at a time. The number of pictures, the rate at which they are displayed and the interstimulus interval can be modified. All target words shown during Word Presentation plus an equal number of decoys will be displayed on the screen one at a time. For each stimulus participants select 'Yes' or 'No' to indicate if they have seen the word before or not. The task outcomes include accuracy and reaction time.

*Delayed Picture Recognition:* A series of pictures are displayed on the screen, one at a time. The number of pictures, the rate at which they are displayed and the interstimulus interval can be modified. All target pictures shown during Picture Presentation plus an equal number of decoys will be displayed on the screen one at a time. For each stimulus participants select 'Yes' or 'No' to indicate if they have seen the picture before or not. The task outcomes include accuracy and reaction time.

## 4. Response to caffeine (positive control)

**Table S3**. Estimated means ± standard error of the mean (SEM) derived from linear mixed models of change from baseline data for outcome measures where the Type III tests of fixed effects showed a significant treatment effect. *p* Values are derived from pairwise comparisons between caffeine and the three other investigational beverages using Sidak adjustment for multiple comparisons.

| | | **Mean** | **SEM** | **p** |
|---|---|---|---|---|
| VAS - Mental Fatigue | Caff | -3.51 | 1.57 | |
| | Pla | 2.84 | 1.58 | <0.001 |
| | 300 mg CB | 4.11 | 1.58 | <0.001 |
| | 100 mg CB | 6.97 | 1.58 | <0.001 |
| VAS - Alertness | Caff | 6.47 | 1.67 | |
| | Pla | -3.78 | 1.68 | <0.001 |
| | 300 mg CB | -1.80 | 1.68 | <0.001 |
| | 100 mg CB | -2.95 | 1.68 | <0.001 |
| VAS - Motivation | Caff | 3.74 | 1.61 | |
| | Pla | -4.56 | 1.62 | <0.001 |
| | 300 mg CB | -5.63 | 1.62 | <0.001 |
| | 100 mg CB | -2.95 | 1.62 | <0.001 |
| EFS - Physical Energy | Caff | 4.62 | 1.90 | |
| | Pla | -1.05 | 1.91 | 0.033 |
| | 300 mg CB | 1.65 | 1.91 | 0.726 |
| | 100 mg CB | -3.73 | 1.92 | 0.002 |
| EFS - Physical Fatigue | Caff | -4.44 | 1.84 | |
| | Pla | 0.52 | 1.85 | 0.123 |
| | 300 mg CB | 1.58 | 1.85 | 0.089 |
| | 100 mg CB | 3.16 | 1.87 | 0.018 |
| EFS - Physical Vigour | Caff | 5.23 | 1.65 | |
| | Pla | 0.03 | 1.66 | 0.019 |
| | 300 mg CB | -0.87 | 1.66 | 0.008 |
| | 100 mg CB | -2.99 | 1.67 | <0.001 |
| EFS - Physical Exhaustion | Caff | -4.62 | 1.90 | |
| | Pla | 0.33 | 1.91 | 0.124 |
| | 300 mg CB | 0.70 | 1.91 | 0.140 |
| | 100 mg CB | 3.74 | 1.92 | 0.003 |
| EFS - Physical Pep | Caff | 5.86 | 1.82 | |
| | Pla | -1.64 | 1.83 | <0.001 |
| | 300 mg CB | -1.09 | 1.83 | 0.006 |
| | 100 mg CB | -3.07 | 1.84 | <0.001 |
| EFS - Physical Worn Out | Caff | -4.67 | 1.77 | |
| | Pla | 0.06 | 1.78 | 0.114 |
| | 300 mg CB | 2.43 | 1.78 | 0.012 |

| | | | | |
|---|---|---|---|---|
| | 100 mg CB | 3.62 | 1.79 | 0.003 |
| EFS - Mental Energy | Caff | 7.77 | 2.02 | |
| | Pla | -1.96 | 2.03 | <0.001 |
| | 300 mg CB | -0.74 | 2.03 | 0.002 |
| | 100 mg CB | -0.90 | 2.04 | 0.002 |
| EFS - Mental Fatigue | Caff | -8.43 | 2.00 | |
| | Pla | 0.75 | 2.01 | <0.001 |
| | 300 mg CB | 0.67 | 2.01 | 0.001 |
| | 100 mg CB | 2.56 | 2.03 | <0.001 |
| EFS - Mental Vigour | Caff | 6.21 | 1.80 | |
| | Pla | -0.33 | 1.80 | 0.002 |
| | 300 mg CB | -0.85 | 1.81 | 0.005 |
| | 100 mg CB | -1.60 | 1.82 | 0.002 |
| EFS - Mental Exhaustion | Caff | -6.58 | 2.05 | |
| | Pla | 2.09 | 2.06 | <0.001 |
| | 300 mg CB | 0.60 | 2.06 | 0.020 |
| | 100 mg CB | 0.65 | 2.07 | 0.024 |
| EFS - Mental Pep | Caff | 7.47 | 1.81 | |
| | Pla | -0.29 | 1.82 | <0.001 |
| | 300 mg CB | -0.80 | 1.82 | 0.001 |
| | 100 mg CB | -2.10 | 1.83 | <0.001 |
| EFS - Mental Worn Out | Caff | -7.35 | 2.00 | |
| | Pla | 0.46 | 2.01 | 0.002 |
| | 300 mg CB | 2.59 | 2.01 | <0.001 |
| | 100 mg CB | 1.26 | 2.02 | 0.004 |
| Bond Lader - Alert | Caff | 8.96 | 1.92 | |
| | Pla | -0.15 | 1.92 | <0.001 |
| | 300 mg CB | 1.27 | 1.93 | 0.001 |
| | 100 mg CB | 0.08 | 1.93 | <0.001 |
| Bond Lader - Content | Caff | 4.24 | 1.17 | |
| | Pla | 0.53 | 1.18 | 0.011 |
| | 300 mg CB | -0.64 | 1.18 | 0.002 |
| | 100 mg CB | 0.99 | 1.19 | 0.109 |
| Serial 3 subtractions Total (number) | Caff | 4.35 | 0.43 | |
| | Pla | 2.26 | 0.43 | <0.001 |
| | 300 mg CB | 1.46 | 0.43 | <0.001 |
| | 100 mg CB | 1.87 | 0.43 | <0.001 |
| Serial 3 subtractions Accuracy (%) | Caff | 0.67 | 0.34 | |
| | Pla | 0.15 | 0.34 | 0.859 |
| | 300 mg CB | -0.39 | 0.34 | 0.149 |
| | 100 mg CB | -0.61 | 0.34 | 0.044 |
| | Caff | 3.61 | 0.29 | |

| | | | | |
|---|---|---|---|---|
| Serial 7 subtractions Total (number) | Pla | 2.60 | 0.29 | 0.013 |
| | 300 mg CB | 2.12 | 0.29 | <0.001 |
| | 100 mg CB | 1.77 | 0.29 | <0.001 |
| Serial 7 subtractions Accuracy (%) | Caff | 1.54 | 0.56 | |
| | Pla | 1.30 | 0.56 | 1.000 |
| | 300 mg CB | -0.24 | 0.57 | 0.093 |
| | 100 mg CB | 0.03 | 0.57 | 0.221 |
| RVIP Accuracy (%) | Caff | 3.85 | 0.79 | |
| | Pla | -2.28 | 0.79 | <0.001 |
| | 300 mg CB | -2.85 | 0.79 | <0.001 |
| | 100 mg CB | -4.38 | 0.79 | <0.001 |
| RVIP RT (ms) | Caff | -8.63 | 2.11 | |
| | Pla | -6.25 | 2.11 | 0.923 |
| | 300 mg CB | -0.15 | 2.13 | 0.006 |
| | 100 mg CB | 0.85 | 2.12 | 0.001 |
| RVIP False alarms (number) | Caff | -0.23 | 0.13 | |
| | Pla | 0.03 | 0.13 | 0.457 |
| | 300 mg CB | 0.28 | 0.14 | 0.009 |
| | 100 mg CB | 0.45 | 0.14 | <0.001 |

Caff, 75 mg caffeine; Pla, placebo; CB coffeeberry; VAS, visual analogue scale; EFS, Energy and Fatigue Scales; RT, reaction time; ms, milliseconds; RVIP, rapid visual information processing

**Table S4.** Performance on the cognitive demand battery task outcomes. The Baseline data are raw means (plus SEM) collected prior to dosing. Data presented from the 60 and 120 minute post dose (p.d.) assessments are estimated means (plus SEM) of change from baseline values derived from the linear mixed model analysis, with the final two columns showing test statistics (F) and associated probabilities (p) for each of the fixed effects included in the model.

| | | | Baseline | | | 60 min p.d. | | | 120 min p.d. | | | | | |
|---|---|---|---|---|---|---|---|---|---|---|---|---|---|---|
| | | | N | Mean | SEM | N | Mean | SEM | N | Mean | SEM | | F | p |
| Serial 3 subtractions Total (number) | Rep 1 | Caff | 70 | 40.97 | 1.69 | 70 | 3.61 | 0.86 | 70 | 4.83 | 0.86 | Treatment | 15.73 | <.001 |
| | | Pla | 69 | 41.52 | 1.67 | | 2.45 | 0.86 | | 1.83 | 0.86 | Treat*Assess | 0.28 | 0.839 |
| | | 300 mg CB | 69 | 42.74 | 1.81 | | 0.58 | 0.87 | | 1.75 | 0.86 | Treat*Rep | 0.45 | 0.909 |
| | | 100 mg CB | 68 | 42.49 | 1.81 | | 1.70 | 0.86 | | 2.31 | 0.86 | Treat*Assess*Rep | 0.74 | 0.713 |
| | Rep 2 | Caff | 70 | 42.01 | 1.78 | | 5.14 | 0.86 | | 4.90 | 0.86 | | | |
| | | Pla | 69 | 41.90 | 1.91 | | 1.52 | 0.87 | | 3.34 | 0.87 | | | |
| | | 300 mg CB | 69 | 44.04 | 1.94 | | 0.73 | 0.87 | | 2.02 | 0.86 | | | |
| | | 100 mg CB | 68 | 42.72 | 1.71 | | 1.51 | 0.86 | | 2.86 | 0.86 | | | |
| | Rep 3 | Caff | 70 | 42.67 | 1.77 | | 3.66 | 0.86 | | 3.49 | 0.86 | | | |
| | | Pla | 68 | 42.28 | 1.84 | | 2.07 | 0.87 | | 0.99 | 0.87 | | | |
| | | 300 mg CB | 69 | 43.32 | 1.88 | | 1.57 | 0.88 | | 1.46 | 0.87 | | | |
| | | 100 mg CB | 68 | 42.43 | 1.69 | | 0.80 | 0.86 | | 2.31 | 0.86 | | | |
| | Rep 4 | Caff | 70 | 41.40 | 1.75 | | 4.73 | 0.86 | | 4.41 | 0.86 | | | |
| | | Pla | 68 | 40.40 | 1.82 | | 2.74 | 0.87 | | 3.16 | 0.87 | | | |
| | | 300 mg CB | 68 | 42.53 | 1.98 | | 1.68 | 0.88 | | 1.92 | 0.87 | | | |
| | | 100 mg CB | 68 | 42.62 | 1.65 | | 1.98 | 0.86 | | 1.48 | 0.86 | | | |
| Serial 3 subtractions Accuracy (%) | Rep 1 | Caff | 70 | 94.74 | 0.70 | 70 | 0.72 | 0.88 | 70 | 0.10 | 0.88 | Treatment | 2.89 | 0.035 |
| | | Pla | 69 | 95.72 | 0.55 | | -1.47 | 0.89 | | -0.26 | 0.89 | Treat*Assess | 0.91 | 0.436 |
| | | 300 mg CB | 69 | 96.12 | 0.64 | | -1.24 | 0.89 | | -1.36 | 0.89 | Treat*Rep | 1.43 | 0.170 |
| | | 100 mg CB | 68 | 95.46 | 0.74 | | 0.32 | 0.89 | | 0.40 | 0.89 | Treat*Assess*Rep | 0.39 | 0.967 |
| | Rep 2 | Caff | 70 | 95.79 | 0.59 | | 0.40 | 0.88 | | 0.12 | 0.88 | | | |
| | | Pla | 69 | 94.78 | 0.73 | | -0.70 | 0.89 | | 0.65 | 0.89 | | | |
| | | 300 mg CB | 69 | 94.96 | 0.68 | | -1.29 | 0.89 | | 0.49 | 0.89 | | | |
| | | 100 mg CB | 68 | 95.13 | 0.75 | | -1.03 | 0.89 | | 0.37 | 0.89 | | | |
| | Rep 3 | Caff | 70 | 95.13 | 0.83 | | 0.74 | 0.88 | | 0.01 | 0.88 | | | |
| | | Pla | 68 | 94.05 | 1.11 | | 0.11 | 0.89 | | 1.18 | 0.89 | | | |
| | | 300 mg CB | 69 | 95.22 | 0.77 | | -0.60 | 0.90 | | -0.72 | 0.89 | | | |
| | | 100 mg CB | 68 | 95.09 | 0.75 | | -1.97 | 0.89 | | -1.24 | 0.89 | | | |
| | Rep 4 | Caff | 70 | 93.66 | 0.92 | | 1.39 | 0.88 | | 1.87 | 0.88 | | | |
| | | Pla | 68 | 93.45 | 1.05 | | 0.57 | 0.89 | | 1.16 | 0.89 | | | |
| | | 300 mg CB | 68 | 92.45 | 1.22 | | -0.28 | 0.90 | | 1.86 | 0.89 | | | |
| | | 100 mg CB | 68 | 95.13 | 0.84 | | -1.58 | 0.89 | | -0.16 | 0.89 | | | |
| Serial 7 subtractions Total (number) | Rep 1 | Caff | 70 | 24.79 | 1.33 | 70 | 2.65 | 0.62 | 70 | 4.87 | 0.61 | Treatment | 11.40 | <.001 |
| | | Pla | 69 | 24.91 | 1.39 | | 2.46 | 0.62 | | 3.77 | 0.62 | Treat*Assess | 0.70 | 0.551 |
| | | 300 mg CB | 69 | 26.39 | 1.45 | | 2.60 | 0.62 | | 2.92 | 0.62 | Treat*Rep | 2.33 | 0.013 |
| | | 100 mg CB | 68 | 25.07 | 1.23 | | 1.55 | 0.62 | | 2.68 | 0.62 | Treat*Assess*Rep | 0.54 | 0.891 |
| | Rep 2 | Caff | 70 | 25.31 | 1.30 | | 2.94 | 0.61 | | 4.77 | 0.61 | | | |

| | | | | | | | | | | | | | | |
|---|---|---|---|---|---|---|---|---|---|---|---|---|---|---|
| | | Pla | 69 | 26.29 | 1.23 | | 2.01 | 0.62 | | 1.89 | 0.62 | | | |
| | | 300 mg CB | 69 | 27.03 | 1.49 | | 1.35 | 0.63 | | 2.09 | 0.62 | | | |
| | | 100 mg CB | 68 | 26.19 | 1.23 | | 1.52 | 0.62 | | 1.76 | 0.62 | | | |
| | Rep 3 | Caff | 70 | 26.40 | 1.31 | | 2.31 | 0.61 | | 3.76 | 0.61 | | | |
| | | Pla | 69 | 25.13 | 1.23 | | 3.01 | 0.62 | | 3.76 | 0.62 | | | |
| | | 300 mg CB | 69 | 26.84 | 1.56 | | 1.93 | 0.62 | | 3.02 | 0.62 | | | |
| | | 100 mg CB | 68 | 26.54 | 1.23 | | 1.02 | 0.62 | | 1.26 | 0.62 | | | |
| | Rep 4 | Caff | 70 | 26.10 | 1.34 | | 3.56 | 0.61 | | 4.03 | 0.61 | | | |
| | | Pla | 68 | 26.69 | 1.37 | | 1.52 | 0.62 | | 2.36 | 0.62 | | | |
| | | 300 mg CB | 69 | 27.54 | 1.55 | | 0.68 | 0.62 | | 2.39 | 0.62 | | | |
| | | 100 mg CB | 68 | 26.25 | 1.27 | | 1.68 | 0.62 | | 2.72 | 0.62 | | | |
| Serial 7 subtractions Accuracy (%) | Rep 1 | Caff | 70 | 90.24 | 1.50 | 70 | 1.43 | 1.39 | 70 | 1.91 | 1.39 | Treatment | 2.91 | 0.034 |
| | | Pla | 69 | 89.24 | 1.46 | | 1.36 | 1.39 | | 3.33 | 1.41 | Treat*Assess | 0.10 | 0.962 |
| | | 300 mg CB | 69 | 92.02 | 1.55 | | 0.92 | 1.41 | | -1.08 | 1.39 | Treat*Rep | 1.23 | 0.272 |
| | | 100 mg CB | 68 | 91.42 | 1.17 | | 0.37 | 1.41 | | -1.08 | 1.41 | Treat*Assess* Rep | 0.73 | 0.726 |
| | Rep 2 | Caff | 70 | 90.90 | 1.32 | | 0.84 | 1.39 | | -0.29 | 1.39 | | | |
| | | Pla | 69 | 89.00 | 1.71 | | 1.21 | 1.39 | | 0.93 | 1.39 | | | |
| | | 300 mg CB | 69 | 92.35 | 1.21 | | -1.68 | 1.42 | | -2.04 | 1.40 | | | |
| | | 100 mg CB | 68 | 90.42 | 1.31 | | -0.04 | 1.41 | | 0.09 | 1.41 | | | |
| | Rep 3 | Caff | 70 | 90.05 | 1.33 | | 0.42 | 1.39 | | 2.07 | 1.39 | | | |
| | | Pla | 69 | 88.53 | 1.39 | | 1.78 | 1.39 | | 2.66 | 1.39 | | | |
| | | 300 mg CB | 69 | 88.99 | 1.50 | | -0.12 | 1.41 | | 1.36 | 1.39 | | | |
| | | 100 mg CB | 68 | 89.89 | 1.40 | | -1.02 | 1.41 | | 0.39 | 1.42 | | | |
| | Rep 4 | Caff | 70 | 87.84 | 1.62 | | 2.38 | 1.39 | | 3.51 | 1.39 | | | |
| | | Pla | 68 | 90.53 | 1.29 | | -0.82 | 1.41 | | -0.08 | 1.40 | | | |
| | | 300 mg CB | 69 | 89.80 | 1.58 | | -1.88 | 1.41 | | 2.62 | 1.39 | | | |
| | | 100 mg CB | 68 | 88.98 | 1.81 | | 0.39 | 1.41 | | 1.10 | 1.41 | | | |
| RVIP Accuracy (%) | Rep 1 | Caff | 69 | 53.55 | 2.64 | 70 | 5.28 | 1.52 | 70 | 1.29 | 1.52 | Treatment | 38.26 | <.001 |
| | | Pla | 69 | 57.25 | 2.66 | | 0.90 | 1.52 | | -6.45 | 1.52 | Treat*Assess | 1.95 | 0.120 |
| | | 300 mg CB | 68 | 53.71 | 2.59 | | 0.99 | 1.54 | | -6.10 | 1.53 | Treat*Rep | 0.47 | 0.895 |
| | | 100 mg CB | 67 | 55.52 | 2.80 | | -1.90 | 1.53 | | -6.56 | 1.54 | Treat*Assess* Rep | 0.60 | 0.845 |
| | Rep 2 | Caff | 69 | 51.67 | 2.67 | | 5.24 | 1.52 | | 1.26 | 1.52 | | | |
| | | Pla | 69 | 53.51 | 2.84 | | 1.05 | 1.52 | | -5.78 | 1.53 | | | |
| | | 300 mg CB | 68 | 50.96 | 2.76 | | 0.05 | 1.54 | | -5.00 | 1.53 | | | |
| | | 100 mg CB | 67 | 53.84 | 2.81 | | -3.14 | 1.53 | | -5.41 | 1.54 | | | |
| | Rep 3 | Caff | 69 | 49.38 | 2.68 | | 5.53 | 1.52 | | 2.05 | 1.52 | | | |
| | | Pla | 69 | 51.41 | 2.77 | | 0.83 | 1.52 | | -5.36 | 1.55 | | | |
| | | 300 mg CB | 68 | 50.77 | 2.80 | | -2.29 | 1.54 | | -5.66 | 1.53 | | | |
| | | 100 mg CB | 67 | 51.38 | 2.79 | | -2.95 | 1.53 | | -5.16 | 1.53 | | | |
| | Rep 4 | Caff | 68 | 47.35 | 2.59 | | 6.51 | 1.53 | | 3.62 | 1.53 | | | |
| | | Pla | 69 | 49.49 | 2.80 | | 0.11 | 1.52 | | -3.58 | 1.54 | | | |
| | | 300 mg CB | 68 | 47.87 | 2.82 | | -1.39 | 1.54 | | -3.41 | 1.54 | | | |
| | | 100 mg CB | 67 | 49.66 | 2.81 | | -4.97 | 1.53 | | -4.94 | 1.53 | | | |
| RVIP RT (ms) | Rep 1 | Caff | 69 | 515.06 | 5.60 | 70 | -2.36 | 4.87 | 70 | -3.91 | 4.87 | Treatment | 6.54 | <.001 |
| | | Pla | 69 | 512.86 | 6.13 | | -0.16 | 4.87 | | 0.81 | 4.87 | Treat*Assess | 0.10 | 0.962 |

| | | | | | | | | | | | | | | |
|---|---|---|---|---|---|---|---|---|---|---|---|---|---|---|
| | | 300 mg CB | 68 | 508.70 | 5.39 | | 4.84 | 4.94 | | 2.17 | 4.91 | Treat*Rep | 1.23 | 0.273 |
| | | 100 mg CB | 67 | 510.54 | 6.88 | | -1.22 | 4.91 | | 1.11 | 4.94 | Treat*Assess* Rep | 0.29 | 0.991 |
| | Rep 2 | Caff | 69 | 527.47 | 6.21 | | -7.24 | 4.87 | | -6.65 | 4.87 | | | |
| | | Pla | 69 | 523.58 | 6.18 | | -7.15 | 4.87 | | -2.72 | 4.91 | | | |
| | | 300 mg CB | 68 | 521.23 | 6.25 | | -4.16 | 4.94 | | -4.64 | 4.91 | | | |
| | | 100 mg CB | 67 | 521.71 | 6.55 | | -3.27 | 4.91 | | -0.28 | 4.94 | | | |
| | Rep 3 | Caff | 69 | 531.86 | 6.53 | | -6.84 | 4.87 | | -13.93 | 4.87 | | | |
| | | Pla | 69 | 531.65 | 6.50 | | -8.65 | 4.87 | | -13.19 | 4.98 | | | |
| | | 300 mg CB | 68 | 522.89 | 6.31 | | 3.11 | 4.94 | | -3.42 | 4.91 | | | |
| | | 100 mg CB | 67 | 520.56 | 5.64 | | 6.86 | 4.91 | | 4.03 | 4.91 | | | |
| | Rep 4 | Caff | 68 | 535.84 | 6.22 | | -13.48 | 4.91 | | -14.66 | 4.91 | | | |
| | | Pla | 69 | 532.16 | 6.28 | | -6.77 | 4.87 | | -12.21 | 4.94 | | | |
| | | 300 mg CB | 68 | 523.36 | 6.91 | | 1.19 | 4.94 | | -0.27 | 4.94 | | | |
| | | 100 mg CB | 67 | 524.05 | 5.95 | | 1.75 | 4.91 | | -2.15 | 4.91 | | | |
| RVIP False alarms (number) | Rep 1 | Caff | 69 | 2.54 | 0.33 | 70 | -0.37 | 0.30 | 70 | -0.19 | 0.30 | Treatment | 6.88 | <.001 |
| | | Pla | 69 | 2.67 | 0.34 | | -0.07 | 0.30 | | -0.30 | 0.30 | Treat*Assess | 0.04 | 0.988 |
| | | 300 mg CB | 68 | 2.37 | 0.40 | | -0.27 | 0.30 | | 0.27 | 0.30 | Treat*Rep | 0.95 | 0.479 |
| | | 100 mg CB | 67 | 2.61 | 0.38 | | -0.12 | 0.30 | | -0.02 | 0.30 | Treat*Assess* Rep | 0.51 | 0.911 |
| | Rep 2 | Caff | 69 | 2.58 | 0.43 | | -0.53 | 0.30 | | -0.50 | 0.30 | | | |
| | | Pla | 69 | 2.68 | 0.40 | | 0.28 | 0.30 | | 0.06 | 0.30 | | | |
| | | 300 mg CB | 68 | 2.12 | 0.35 | | 0.58 | 0.30 | | 0.37 | 0.30 | | | |
| | | 100 mg CB | 67 | 2.19 | 0.30 | | 0.49 | 0.30 | | 0.55 | 0.30 | | | |
| | Rep 3 | Caff | 69 | 2.22 | 0.28 | | -0.06 | 0.30 | | 0.21 | 0.30 | | | |
| | | Pla | 69 | 2.65 | 0.42 | | 0.01 | 0.30 | | 0.30 | 0.31 | | | |
| | | 300 mg CB | 68 | 2.19 | 0.35 | | 0.69 | 0.30 | | 0.47 | 0.30 | | | |
| | | 100 mg CB | 67 | 2.21 | 0.34 | | 0.71 | 0.30 | | 0.78 | 0.30 | | | |
| | Rep 4 | Caff | 68 | 2.65 | 0.39 | | -0.06 | 0.30 | | -0.33 | 0.30 | | | |
| | | Pla | 69 | 2.77 | 0.37 | | -0.18 | 0.30 | | 0.18 | 0.30 | | | |
| | | 300 mg CB | 68 | 2.63 | 0.56 | | -0.14 | 0.30 | | 0.27 | 0.30 | | | |
| | | 100 mg CB | 67 | 2.45 | 0.29 | | 0.46 | 0.30 | | 0.75 | 0.30 | | | |
| Mental Fatigue | Rep 1 | Caff | 72 | 38.24 | 2.20 | 72 | -2.14 | 2.47 | 72 | 7.51 | 2.47 | Treatment | 15.43 | <.001 |
| | | Pla | 71 | 42.23 | 2.01 | | -0.78 | 2.49 | | 8.80 | 2.49 | Treat*Assess | 0.74 | 0.527 |
| | | 300 mg CB | 71 | 38.56 | 2.09 | | 5.89 | 2.50 | | 10.26 | 2.49 | Treat*Rep | 1.92 | 0.045 |
| | | 100 mg CB | 70 | 37.97 | 2.00 | | 7.67 | 2.49 | | 11.92 | 2.49 | Treat*Assess* Rep | 1.42 | 0.148 |
| | Rep 2 | Caff | 72 | 48.26 | 2.32 | | -7.17 | 2.47 | | -3.01 | 2.47 | | | |
| | | Pla | 71 | 46.49 | 2.19 | | 1.19 | 2.49 | | 9.25 | 2.49 | | | |
| | | 300 mg CB | 71 | 46.01 | 2.19 | | 3.30 | 2.50 | | 8.18 | 2.49 | | | |
| | | 100 mg CB | 70 | 43.94 | 2.15 | | 6.87 | 2.49 | | 11.52 | 2.49 | | | |
| | Rep 3 | Caff | 72 | 52.43 | 2.38 | | -7.01 | 2.47 | | -2.15 | 2.47 | | | |
| | | Pla | 71 | 51.01 | 2.14 | | 0.66 | 2.49 | | 7.01 | 2.49 | | | |
| | | 300 mg CB | 71 | 51.41 | 2.31 | | 2.25 | 2.50 | | 5.70 | 2.49 | | | |
| | | 100 mg CB | 70 | 48.93 | 2.29 | | 6.22 | 2.49 | | 6.63 | 2.49 | | | |
| | Rep 4 | Caff | 72 | 54.97 | 2.33 | | -8.53 | 2.47 | | -5.58 | 2.47 | | | |
| | | Pla | 71 | 55.66 | 2.04 | | -7.05 | 2.49 | | 3.68 | 2.49 | | | |

| | | | | | | | | | | | | | | |
|---|---|---|---|---|---|---|---|---|---|---|---|---|---|---|
| | | 300 mg CB | 71 | 55.42 | 2.40 | | -6.77 | 2.50 | | 4.05 | 2.49 | | | |
| | | 100 mg CB | 70 | 52.64 | 2.38 | | -2.56 | 2.49 | | 7.49 | 2.49 | | | |
| | Rep 1 | Caff | 72 | 52.43 | 2.00 | 72 | 5.15 | 2.49 | 72 | -1.88 | 2.49 | Treatment | 21.73 | <.001 |
| | | Pla | 71 | 52.82 | 2.01 | | -0.06 | 2.50 | | -9.11 | 2.50 | Treat*Assess | 2.14 | 0.094 |
| | | 300 mg CB | 71 | 53.30 | 2.00 | | -1.50 | 2.51 | | -8.68 | 2.50 | Treat*Rep | 1.62 | 0.105 |
| | | 100 mg CB | 70 | 50.51 | 2.41 | | -0.17 | 2.50 | | -5.35 | 2.50 | Treat*Assess* Rep | 0.53 | 0.899 |
| | Rep 2 | Caff | 72 | 45.46 | 2.06 | | 8.78 | 2.49 | | 2.56 | 2.49 | | | |
| | | Pla | 71 | 48.86 | 2.19 | | -1.53 | 2.50 | | -8.89 | 2.50 | | | |
| | | 300 mg CB | 71 | 47.93 | 2.08 | | -1.08 | 2.52 | | -4.93 | 2.50 | | | |
| Alertness | | 100 mg CB | 70 | 47.24 | 2.37 | | -3.66 | 2.50 | | -5.13 | 2.50 | | | |
| | Rep 3 | Caff | 72 | 39.92 | 2.11 | | 13.51 | 2.49 | | 5.88 | 2.49 | | | |
| | | Pla | 71 | 43.52 | 2.20 | | -0.13 | 2.50 | | -7.57 | 2.50 | | | |
| | | 300 mg CB | 71 | 42.72 | 2.31 | | -0.92 | 2.52 | | -1.79 | 2.50 | | | |
| | | 100 mg CB | 70 | 43.71 | 2.45 | | -3.54 | 2.50 | | -2.74 | 2.50 | | | |
| | Rep 4 | Caff | 72 | 38.85 | 2.08 | | 11.07 | 2.49 | | 6.69 | 2.49 | | | |
| | | Pla | 71 | 39.83 | 1.98 | | 1.22 | 2.50 | | -4.16 | 2.50 | | | |
| | | 300 mg CB | 71 | 37.94 | 2.26 | | 2.43 | 2.51 | | 2.04 | 2.50 | | | |
| | | 100 mg CB | 70 | 40.77 | 2.44 | | -0.88 | 2.50 | | -2.14 | 2.50 | | | |
| | Rep 1 | Caff | 72 | 53.00 | 1.90 | 72 | 2.43 | 2.19 | 72 | -4.18 | 2.19 | Treatment | 21.46 | <.001 |
| | | Pla | 71 | 54.10 | 2.07 | | -2.00 | 2.20 | | -9.23 | 2.20 | Treat*Assess | 0.32 | 0.808 |
| | | 300 mg CB | 71 | 56.41 | 1.74 | | -3.60 | 2.21 | | -12.90 | 2.20 | Treat*Rep | 1.74 | 0.076 |
| | | 100 mg CB | 70 | 52.26 | 2.26 | | -3.17 | 2.20 | | -8.10 | 2.20 | Treat*Assess* Rep | 0.91 | 0.531 |
| | Rep 2 | Caff | 72 | 47.10 | 2.15 | | 6.18 | 2.19 | | 0.39 | 2.19 | | | |
| | | Pla | 71 | 49.72 | 2.20 | | -3.13 | 2.20 | | -8.79 | 2.20 | | | |
| | | 300 mg CB | 71 | 52.42 | 1.98 | | -5.77 | 2.21 | | -11.85 | 2.20 | | | |
| Motivation | | 100 mg CB | 70 | 47.47 | 2.22 | | -1.31 | 2.20 | | -6.75 | 2.20 | | | |
| | Rep 3 | Caff | 72 | 43.26 | 2.26 | | 7.74 | 2.19 | | 2.51 | 2.19 | | | |
| | | Pla | 71 | 47.13 | 2.01 | | -2.18 | 2.20 | | -8.66 | 2.20 | | | |
| | | 300 mg CB | 71 | 46.41 | 2.23 | | -3.20 | 2.21 | | -6.65 | 2.20 | | | |
| | | 100 mg CB | 70 | 43.03 | 2.30 | | -1.69 | 2.20 | | -1.79 | 2.20 | | | |
| | Rep 4 | Caff | 72 | 39.43 | 2.22 | | 10.08 | 2.19 | | 4.75 | 2.19 | | | |
| | | Pla | 71 | 40.59 | 2.07 | | 0.77 | 2.20 | | -3.28 | 2.20 | | | |
| | | 300 mg CB | 71 | 41.21 | 2.18 | | 1.31 | 2.21 | | -2.41 | 2.20 | | | |
| | | 100 mg CB | 70 | 40.33 | 2.38 | | 2.38 | 2.20 | | -3.14 | 2.20 | | | |

Caff, 75 mg caffeine; Pla, placebo; CB coffeeberry; VAS, visual analogue scale; EFS, Energy and Fatigue Scales; RT, reaction time; ms, milliseconds; RVIP, rapid visual information processing; Treat, treatment; Assess, assessment; Rep, repetition

**Table S5.** Scores for the Energy and Fatigue and Bond-Lader mood scales. The Baseline data are raw means (plus SEM) collected prior to dosing. Data presented from the 60 and 120 minute post dose (p.d.) assessments are estimated means (plus SEM) of change from baseline values derived from the linear mixed model analysis, with the final two columns showing test statistics (F) and associated probabilities (p) for each of the fixed effects included in the model.

| | | Baseline | | | 60 min p.d. | | | 120 min p.d. | | | | | |
|---|---|---|---|---|---|---|---|---|---|---|---|---|---|
| | | N | Mean | SEM | N | Mean | SEM | N | Mean | SEM | | F | p |
| Physical Energy | Caff | 72 | 47.92 | 2.03 | 72 | 5.64 | 2.22 | 72 | 3.60 | 2.22 | Treatment | 5.96 | <.001 |
| | Pla | 71 | 46.18 | 1.93 | | 2.12 | 2.23 | | -4.21 | 2.23 | Treat*Assess | 0.85 | 0.467 |
| | 300 mg CB | 71 | 46.21 | 2.26 | | 2.23 | 2.23 | | 1.08 | 2.23 | | | |
| | 100 mg CB | 70 | 47.66 | 2.05 | | -2.25 | 2.24 | | -5.21 | 2.24 | | | |
| Physical Fatigue | Caff | 72 | 46.07 | 2.12 | 72 | -7.22 | 2.19 | 72 | -1.67 | 2.19 | Treatment | 3.45 | 0.017 |
| | Pla | 71 | 46.56 | 1.79 | | -2.60 | 2.20 | | 3.64 | 2.20 | Treat*Assess | 0.53 | 0.659 |
| | 300 mg CB | 71 | 45.00 | 2.07 | | 0.03 | 2.20 | | 3.14 | 2.20 | | | |
| | 100 mg CB | 70 | 46.23 | 2.08 | | 1.72 | 2.21 | | 4.59 | 2.22 | | | |
| Physical Vigour | Caff | 72 | 44.21 | 1.81 | | 6.85 | 1.96 | | 3.61 | 1.96 | Treatment | 6.83 | <.001 |
| | Pla | 71 | 43.25 | 1.77 | 72 | 3.14 | 1.97 | 72 | -3.09 | 1.98 | Treat*Assess | 1.43 | 0.235 |
| | 300 mg CB | 71 | 45.31 | 2.11 | | -0.83 | 1.98 | | -0.91 | 1.98 | | | |
| | 100 mg CB | 70 | 45.66 | 2.03 | | -2.12 | 1.99 | | -3.85 | 1.99 | | | |
| Physical Exhaustion | Caff | 72 | 45.44 | 2.17 | 72 | -5.74 | 2.28 | 72 | -3.50 | 2.28 | Treatment | 4.33 | 0.005 |
| | Pla | 71 | 47.94 | 1.94 | | -1.37 | 2.29 | | 2.03 | 2.29 | Treat*Assess | 0.10 | 0.961 |
| | 300 mg CB | 71 | 45.41 | 2.25 | | 0.00 | 2.29 | | 1.39 | 2.29 | | | |
| | 100 mg CB | 70 | 45.01 | 1.90 | | 2.38 | 2.30 | | 5.09 | 2.31 | | | |
| Physical Pep | Caff | 72 | 43.00 | 2.07 | 72 | 7.19 | 2.11 | 72 | 4.53 | 2.11 | Treatment | 7.86 | <.001 |
| | Pla | 71 | 42.18 | 1.88 | | 0.39 | 2.12 | | -3.66 | 2.12 | Treat*Assess | 0.32 | 0.812 |
| | 300 mg CB | 71 | 42.96 | 2.16 | | -0.60 | 2.12 | | -1.58 | 2.12 | | | |
| | 100 mg CB | 70 | 44.74 | 2.07 | | -1.88 | 2.13 | | -4.25 | 2.13 | | | |
| Physical Worn Out | Caff | 72 | 45.90 | 2.24 | 72 | -6.46 | 2.10 | 72 | -2.88 | 2.10 | Treatment | 4.85 | 0.003 |
| | Pla | 71 | 48.45 | 1.97 | | -1.44 | 2.12 | | 1.55 | 2.12 | Treat*Assess | 0.20 | 0.896 |
| | 300 mg CB | 71 | 46.41 | 2.09 | | 1.82 | 2.12 | | 3.04 | 2.12 | | | |
| | 100 mg CB | 70 | 45.09 | 2.03 | | 2.49 | 2.13 | | 4.74 | 2.13 | | | |
| Mental Energy | Caff | 72 | 39.32 | 1.84 | 72 | 10.75 | 2.33 | 72 | 4.79 | 2.33 | Treatment | 8.49 | <.001 |
| | Pla | 71 | 38.87 | 1.75 | | 1.01 | 2.34 | | -4.93 | 2.34 | Treat*Assess | 0.49 | 0.690 |
| | 300 mg CB | 71 | 38.83 | 2.01 | | 1.72 | 2.34 | | -3.20 | 2.34 | | | |
| | 100 mg CB | 70 | 38.00 | 2.06 | | 0.37 | 2.36 | | -2.16 | 2.36 | | | |
| Mental Fatigue | Caff | 72 | 54.21 | 2.03 | 72 | -11.33 | 2.32 | 72 | -5.53 | 2.32 | Treatment | 8.89 | <.001 |
| | Pla | 71 | 54.27 | 1.69 | | -3.13 | 2.33 | | 4.63 | 2.33 | Treat*Assess | 0.18 | 0.913 |
| | 300 mg CB | 71 | 54.87 | 1.86 | | -2.11 | 2.33 | | 3.44 | 2.33 | | | |
| | 100 mg CB | 70 | 54.19 | 1.95 | | -0.33 | 2.35 | | 5.45 | 2.35 | | | |
| Mental Vigour | Caff | 72 | 40.61 | 1.76 | 72 | 8.35 | 2.05 | 72 | 4.07 | 2.05 | Treatment | 6.20 | <.001 |
| | Pla | 71 | 39.52 | 1.52 | | 2.93 | 2.06 | | -3.60 | 2.06 | Treat*Assess | 0.84 | 0.473 |
| | 300 mg CB | 71 | 39.52 | 1.90 | | 0.30 | 2.06 | | -2.01 | 2.06 | | | |
| | 100 mg CB | 70 | 40.71 | 1.95 | | -0.13 | 2.07 | | -3.08 | 2.08 | | | |
| Mental Exhaustion | Caff | 72 | 53.33 | 2.03 | 72 | -8.81 | 2.35 | 72 | -4.36 | 2.35 | Treatment | 6.16 | <.001 |
| | Pla | 71 | 54.76 | 1.88 | | -1.26 | 2.36 | | 5.43 | 2.36 | Treat*Assess | 0.21 | 0.887 |

| | | | | | | | | | | | | | |
|---|---|---|---|---|---|---|---|---|---|---|---|---|---|
| | 300 mg CB | 71 | 54.96 | 2.03 | | -2.41 | 2.36 | | 3.62 | 2.36 | | | |
| | 100 mg CB | 70 | 55.33 | 1.99 | | -1.62 | 2.38 | | 2.93 | 2.38 | | | |
| Mental Pep | Caff | 72 | 38.36 | 1.83 | 72 | 8.89 | 2.09 | 72 | 6.06 | 2.09 | Treatment | 9.02 | <.001 |
| | Pla | 71 | 37.41 | 1.64 | | 2.55 | 2.10 | | -3.12 | 2.11 | Treat*Assess | 0.35 | 0.789 |
| | 300 mg CB | 71 | 38.68 | 1.93 | | 0.67 | 2.11 | | -2.27 | 2.11 | | | |
| | 100 mg CB | 70 | 38.46 | 1.96 | | -0.39 | 2.12 | | -3.81 | 2.12 | | | |
| Mental Worn Out | Caff | 72 | 52.74 | 2.22 | 72 | -9.32 | 2.32 | 72 | -5.38 | 2.32 | Treatment | 6.71 | <.001 |
| | Pla | 71 | 54.99 | 1.71 | | -2.78 | 2.34 | | 3.71 | 2.34 | Treat*Assess | 0.25 | 0.862 |
| | 300 mg CB | 71 | 53.92 | 2.08 | | 0.34 | 2.34 | | 4.84 | 2.34 | | | |
| | 100 mg CB | 70 | 54.03 | 2.08 | | -0.64 | 2.35 | | 3.16 | 2.35 | | | |
| Bond Lader - Alert | Caff | 72 | 43.62 | 1.87 | 72 | 10.58 | 2.13 | 72 | 7.33 | 2.13 | Treatment | 11.49 | <.001 |
| | Pla | 71 | 44.10 | 1.78 | | 2.26 | 2.14 | | -2.56 | 2.14 | Treat*Assess | 0.83 | 0.477 |
| | 300 mg CB | 71 | 43.44 | 2.05 | | 1.70 | 2.14 | | 0.83 | 2.14 | | | |
| | 100 mg CB | 70 | 42.91 | 2.01 | | 0.96 | 2.15 | | -0.80 | 2.15 | | | |
| Bond Lader - Content | Caff | 72 | 58.02 | 1.90 | 72 | 5.01 | 1.34 | 72 | 3.47 | 1.34 | Treatment | 4.95 | 0.002 |
| | Pla | 71 | 57.15 | 1.80 | | 1.98 | 1.35 | | -0.92 | 1.35 | Treat*Assess | 1.03 | 0.380 |
| | 300 mg CB | 71 | 58.53 | 1.87 | | -0.79 | 1.35 | | -0.50 | 1.35 | | | |
| | 100 mg CB | 70 | 57.37 | 1.78 | | 1.32 | 1.36 | | 0.66 | 1.36 | | | |
| Bond Lader - Calm | Caff | 72 | 63.81 | 1.54 | 72 | -4.79 | 1.51 | 72 | -4.37 | 1.51 | Treatment | 2.15 | 0.094 |
| | Pla | 71 | 63.29 | 1.59 | | -1.57 | 1.52 | | -2.71 | 1.52 | Treat*Assess | 0.19 | 0.904 |
| | 300 mg CB | 71 | 63.94 | 1.70 | | -2.27 | 1.52 | | -3.16 | 1.52 | | | |
| | 100 mg CB | 70 | 64.50 | 1.59 | | -0.67 | 1.53 | | -0.92 | 1.53 | | | |

Caff, 75 mg caffeine; Pla, placebo; CB coffeeberry

**Table S6.** Scores for the episodic memory tasks, completed following the 120 minute post dose assessment. Data are estimated means (plus SEM) derived from the linear mixed model analysis, with the final two columns showing test statistics (F) and associated probabilities (p) for the effect of treatment.

| | | N | Mean | SEM | F | p |
|---|---|---|---|---|---|---|
| Word recognition Accuracy (%) | Caff | 72 | 68.75 | 1.26 | 1.35 | 0.258 |
| | Pla | | 69.41 | 1.27 | | |
| | 300 mg CB | | 68.66 | 1.27 | | |
| | 100 mg CB | | 66.69 | 1.27 | | |
| Word recognition RT (ms) | Caff | 72 | 1063.36 | 29.97 | 1.67 | 0.174 |
| | Pla | | 1017.04 | 30.10 | | |
| | 300 mg CB | | 1068.40 | 30.10 | | |
| | 100 mg CB | | 1036.36 | 30.20 | | |
| Picture recognition Accuracy (%) | Caff | 72 | 79.91 | 1.65 | 0.08 | 0.970 |
| | Pla | | 79.75 | 1.66 | | |
| | 300 mg CB | | 80.12 | 1.66 | | |
| | 100 mg CB | | 79.44 | 1.67 | | |
| Picture recognition RT (ms) | Caff | 72 | 926.10 | 21.32 | 0.84 | 0.473 |
| | Pla | | 900.22 | 21.42 | | |
| | 300 mg CB | | 922.78 | 21.42 | | |
| | 100 mg CB | | 907.65 | 21.57 | | |
| Delayed word recall Number | Caff | 72 | 2.22 | 0.20 | 1.20 | 0.312 |
| | Pla | | 2.56 | 0.20 | | |
| | 300 mg CB | | 2.20 | 0.20 | | |
| | 100 mg CB | | 2.31 | 0.20 | | |

**References**


1. Kennedy, D.O.; Haskell, C.F.; Robertson, B.; Reay, J.; Brewster-Maund, C.; Luedemann, J.; Maggini, S.; Ruf, M.; Zangara, A.; Scholey, A.B. Improved cognitive performance and mental fatigue following a multi-vitamin and mineral supplement with added guarana (Paullinia cupana). *Appetite* **2008**, *50*, 506-513, doi:10.1016/j.appet.2007.10.007.
2. Kennedy, D.O.; Scholey, A.B. A glucose-caffeine 'energy drink' ameliorates subjective and performance deficits during prolonged cognitive demand. *Appetite* **2004**, *42*, 331-333, doi:10.1016/j.appet.2004.03.001.
3. Reay, J.L.; Kennedy, D.O.; Scholey, A.B. Single doses of Panax ginseng (G115) reduce blood glucose levels and improve cognitive performance during sustained mental activity. *J Psychopharmacol* **2005**, *19*, 357-365, doi:10.1177/0269881105053286.
4. Reay, J.L.; Kennedy, D.O.; Scholey, A.B. Effects of Panax ginseng, consumed with and without glucose, on blood glucose levels and cognitive performance during sustained 'mentally demanding' tasks. *J Psychopharmacol* **2006**, *20*, 771-781, doi:10.1177/0269881106061516.
5. Kennedy, D.; Okello, E.; Chazot, P.; Howes, M.-J.; Ohiomokhare, S.; Jackson, P.; Haskell-Ramsay, C.; Khan, J.; Forster, J.; Wightman, E. Volatile terpenes and brain function: Investigation of the cognitive and mood effects of Mentha× piperita l. essential oil with in vitro properties relevant to central nervous system function. *Nutrients* **2018**, *10*, 1029.
6. Scholey, A.B.; French, S.J.; Morris, P.J.; Kennedy, D.O.; Milne, A.L.; Haskell, C.F. Consumption of cocoa flavanols results in acute improvements in mood and cognitive performance during sustained mental effort. *J Psychopharmacol* **2010**, *24*, 1505-1514, doi:10.1177/0269881109106923.
